# Moving south: effects of water temperatures on the larval development of invasive cane toads (*Rhinella marina*) in cool‐temperate Australia

**DOI:** 10.1002/ece3.2405

**Published:** 2016-09-09

**Authors:** Uditha Wijethunga, Matthew Greenlees, Richard Shine

**Affiliations:** ^1^ School of Life and Environmental Sciences University of Sydney Sydney NSW 2006 Australia

**Keywords:** amphibian, *Bufo marinus*, development, invasive species, physiological tolerance, temperature

## Abstract

The distributional limits of many ectothermic species are set by thermal tolerances of early‐developmental stages in the life history; embryos and larvae often are less able to buffer environmental variation than are conspecific adults. In pond‐breeding amphibians, for example, cold water may constrain viability of eggs and larvae, even if adults can find suitable thermal conditions in terrestrial niches. Invasive species provide robust model systems for exploring these questions, because we can quantify thermal challenges at the expanding range edge (from field surveys) and larval responses to thermal conditions (in the laboratory). Our studies on invasive cane toads (*Rhinella marina*) at the southern (cool‐climate) edge of their expanding range in Australia show that available ponds often average around 20°C during the breeding period, 10°C lower than in many areas of the toads’ native range, or in the Australian tropics. Our laboratory experiments showed that cane toad eggs and larvae cannot develop successfully at 16°C, but hatching success and larval survival rates were higher at 20°C than in warmer conditions. Lower temperatures slowed growth rates, increasing the duration of tadpole life, but also increased metamorph body mass. Water temperature also influenced metamorph body shape (high temperatures reduced relative limb length, head width, and body mass) and locomotor performance (increased speed from intermediate temperatures, longer hops from high temperatures). In combination with previous studies, our data suggest that lower water temperatures may enhance rather than reduce recruitment of cane toads, at least in areas where pond temperatures reach or exceed 20°C. That condition is fulfilled over a wide area of southern Australia, suggesting that the continuing expansion of this invasive species is unlikely to be curtailed by the impacts of relatively low water temperatures on the viability of early life‐history stages.

## 1 Introduction

The limits to geographic distribution of many species are set by environmental temperatures, with far fewer taxa in cool‐climate habitats than in warmer ones (Buckley & Jetz, 2007; Muth, 1980; Southward, 1958). As world climates have warmed, many species have expanded their ranges into areas that were previously too cool for them (Gibbons et al., 2000; Hughes, Cawsey, & Westoby, 1996a,b). The proximate mechanisms that prevent a given species from extending into cooler areas have been elucidated in several taxa (e.g., Hakam & Simon, 2000; Roy, Simon, & Lapointe, 2000), but remain unclear in many cases. Commonly, ontogenetic variation in sensitivity to low temperatures may be important: in which case distributional limits will be set by the vulnerability of the life‐history stages that are most sensitive (Beaty & Salice, 2013; Crouse, Crowder, & Caswell, 1987; Muth, 1980; Packard & Packard, 1988). For example, an ability to thermoregulate behaviorally (select thermally favorable habitats) allows terrestrial squamate reptiles to survive in severely cold habitats, but the embryos of such species cannot move around to escape lethally low soil temperatures, constraining oviparous reptiles to relatively warm climates (Shine, 1987; Shine, Elphick, & Barrott, 2003). The evolution of viviparity removes this constraint, by allowing reproducing females to maintain high temperatures for their developing embryos, and thus, viviparous species can exploit much colder habitats than can their egg‐laying relatives (Qualls, Shine, Donnellan, & Hutchinson, 1995; Shine, 2002, 2005).

Pond‐breeding amphibians could reasonably be expected to exhibit similar ontogenetic shifts in sensitivity to environmental conditions. The eggs and larvae of such species are restricted to their natal pond, and thus must develop under the conditions in that water body. Conspecific adults can move across the landscape to select optimal microhabitats, but aquatic life‐history stages (eggs, larvae) have little or no opportunity to escape the conditions (of temperature, pH, salinity, etc.) in the pond in which they develop. As a result, the physiological tolerance of amphibian eggs and larvae to abiotic factors (e.g., the impact of water temperature on development, growth, and hatching success) may constrain geographic distributions, preventing a species from extending into areas without aquatic habitats suitable for larvae (even if those sites provide suitable conditions for terrestrial adults).

These issues have direct management implications for any attempt to predict (and potentially, limit) the expansion of invasive anuran species. Taxa such as American bullfrogs (*Lithobates catesbeiana*), African clawed frogs (*Xenopus laevis*), and cane toads (*Rhinella* [*Bufo*] *marina*) can severely impact native biota (Kiesecker & Blaustein, 1998; Lafferty & Page, 1997; Letnic, Webb, & Shine, 2008; Pearl, Adams, Bury, & McCreary, 2004; Phillips, Brown, & Shine, 2003). Attempts to predict the dynamics of the invasion process and eventual distribution of such taxa in their invaded range are fraught with difficulty, with alternative methods providing very divergent predictions (Bolzoni, Pugliese, & Rosà, 2015; Phillips, 2015; Shine & Phillips, 2014). The most robust basis for prediction will come from a combination of field surveys (documenting conditions in available water bodies) and laboratory experiments (exploring impacts of water body conditions on invader viability). Given the ability of some invasive taxa to evolve rapidly in response to local conditions (Colautti & Barrett, 2013; Ghalambor, McKay, Carroll, & Reznick, 2007; Huey, Gilchrist, Carlson, Berrigan, & Serra, 2000; Williams & Moore, 1989), such studies need to use animals sourced from the locations of interest, rather than elsewhere within the species’ range (Bossdorf et al., 2005).

We have conducted such a study on invasive cane toads, at the southern (cool‐climate) edge of the species’ expanding range in Australia. Most research on this species has been conducted in the tropics, where the toad invasion front is expanding rapidly (>50 km/year; Phillips, Brown, Webb, & Shine, 2006). However, cane toads are also spreading along the southeastern coast of Australia, with the leading edge of the invasion currently located in northeastern New South Wales (NSW; Seabrook, 1991). The climate in this region is cooler than over most of the species’ native range, or its invaded range within Australia (McCann, Greenlees, Newell, & Shine, 2014). Previous studies have documented major geographic differentiation in phenotypic traits among Australian population of toads (e.g., Brown, Phillips, Dubey, & Shine, 2015; Llewelyn, Phillips, Alford, Schwarzkopf, & Shine, 2010; Phillips et al., 2006). Given this evidence for rapid phenotypic evolution, we might expect higher cold tolerance at the expanding edge of the southern invasion front than in other areas. We conducted field surveys of available spawning sites to quantify thermal conditions and used laboratory experiments to investigate the consequences of those conditions for the viability of toad eggs, for rates of larval growth and developmental rate, for the duration of larval life, and for the size, morphology, and locomotor ability of metamorphs.

## 2 Material and Methods

### 2.1 Study species and area

**Figure 1:**
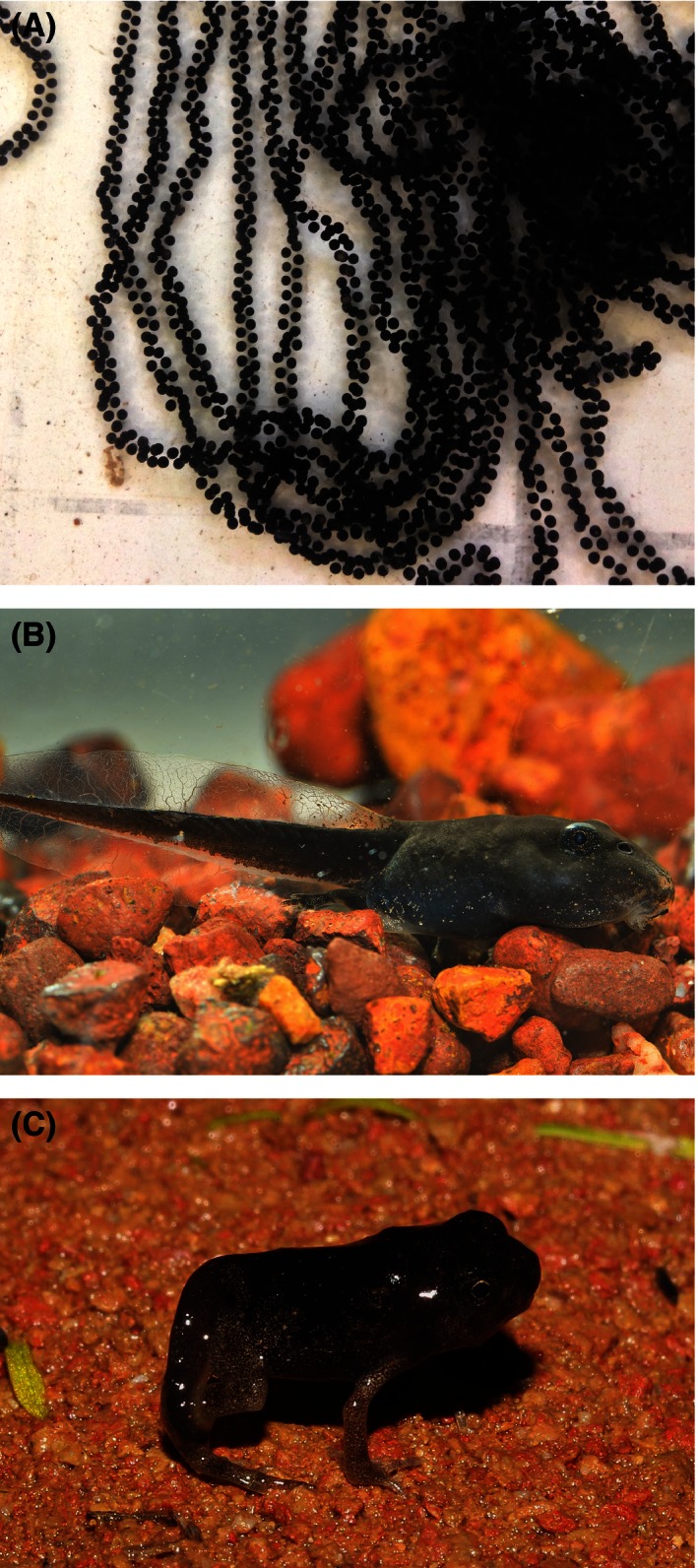
Photographs of early life‐history stages of the cane toad *Rhinella marina*: (A) eggs, (B) tadpole, and (C) metamorph. Photographs by (A) R. Shine, (B) G. Clarke, and (C) M. Greenlees

Native to Mexico, Central America, and South America, the cane toad has been translocated to many countries outside its native range (Lever, 2001). These large anurans (adults typically around 100–400 g body mass; Beckmann & Shine, 2011; Kelehear, Brown, & Shine, 2011) prefer disturbed habitats modified by human activity (Zug & Zug, 1979). Cane toads were introduced to Australia in 1935 in an unsuccessful attempt to control sugarcane beetles (Lever, 2001) and have spread rapidly through the Australian tropics (northern areas of Queensland, the Northern Territory, and Western Australia; Phillips, Brown, Greenlees, Webb, & Shine, 2007; Shine, 2010). They have also spread south into cooler areas of northern NSW (McCann et al., 2014). Cane toads breed in shallow pools (Hagman & Shine, 2006), laying up to 30,000 eggs per clutch (Zug & Zug, 1979). Eggs hatch within 1–2 days into small black tadpoles that develop through to metamorphic stage in a few weeks (Fig. 1). Because cane toads generally spawn in ponds that are sun‐exposed rather than sheltered by dense vegetation, water temperatures are high (Semeniuk, Lemckert, & Shine, 2007). In the Australian tropics, Hagman and Shine (2006) found a mean temperature of around 32°C in spawning ponds, similar to that in adjacent ponds not used for breeding. In a comparable study conducted on cane toads in southern Australia, Semeniuk et al. (2007) reported that toads bred in ponds with an average temperature of 28.5°C, significantly higher than the mean temperature (26.9°C) in adjacent ponds not used as spawning sites.

To investigate thermal conditions in potential breeding ponds even further south, near the slowly expanding southern edge of the cane toads’ Australian range, we measured water temperatures at available spawning sites (based on the presence of toad eggs and/or tadpoles) in 28 ponds in northeastern NSW (centered on 33°56′38.69″S, 147°56′57.41″E). The southeastern edge of the cane toad invasion is moving through a relatively narrow corridor of low‐lying land between the Pacific Ocean (to the east) and the mountains of the Great Dividing Range (to the west). The available breeding sites range from roadside culverts to large water‐retention dams on farms.

### 2.2 Measurements of water temperatures in spawning ponds

We sampled water bodies in the spring (September–October 2012 and 2013) and autumn (March 2013), to encompass the range of water temperatures at times of year when ambient temperatures are relatively low, but toad eggs and tadpoles can be found in ponds. These ponds doubtless are warmer in midsummer (Semeniuk et al., 2007), but the critical issue for the continuing southern invasion of toads is their ability to develop successfully at temperatures that are close to the lower extremes now encountered during the breeding season. That is, ponds south of the toad's current range are likely to exhibit water temperatures in midsummer that are similar to those shown in spring and autumn at the current range edge. We thus measured water temperatures in spring and autumn, not summer. All surveys were conducted between 0900 and 2000 hr, using four thermal data loggers (Thermochron iButton model DS1921G‐F5; Dallas Semiconductor, USA, immersed at 20–45 cm deep near the shallow edge of the pond) in each water body, with each logger recording data once per hour for 5 days (accuracy ±1°C). Temperatures were recorded in 28 ponds. We collected data from five of these ponds at all three time periods (September–October 2012 and 2013, and March 2013), and for the other 23 in one time period only (due to pond drying).

### 2.3 Experimental study

We captured adult toads from the ponds in which we measured temperatures, as well as from the margins of adjacent water bodies (all near Yamba, Northern NSW). These animals were housed at the University of Sydney for 3–6 months (in large plastic bins with ad libitum access to water and shelter, fed twice weekly with crickets). We obtained eggs by injecting pairs of toads with synthetic gonadotropins (0.5 ml/L, Lucrin, Abbott Australasia, Botany, NSW; see Kelehear, Webb, & Shine, 2009 for details). The resulting fertilized eggs (of two clutches) were separated into strands each containing 100 eggs (Gosner stage 12–15; Gosner, 1960), such that at each temperature treatment we had three strands from each clutch (and thus in total, six replicates for each treatment temperature). Each strand of eggs was placed in a tub (28 × 38 × 20 cm) containing unchlorinated water (API Tap Water Conditioner, Chalfont, Pasadena; 5 ml per 20 L) that was kept at one of five different temperatures (16, 20, 24, 28, and 32°C). Each thermal treatment was replicated six times. The temperature of water in tubs was maintained using aquarium heaters (AquaWorld, Model HT 2150; Boyu Group, Guangdong, China) and recorded daily (EC‐PCST Testr35 multiparameter, model PTTEST35, Eutech, Singapore; accuracy ±0.5°C). The water was aerated every day for 15 min each day with a 220‐ to 240‐V aerator (Resun LP‐100; Longgang, Shenzhen, China), pumping through a 28.4‐mm cylindrical air stone (A960 Marina Air Stone; Hagen, Baie‐d'Urfé, Quebec). The numbers of hatched eggs (Gosner stage 18–20) were recorded after 2 days of exposure to these thermal treatments. At this time, all eggs had either hatched or were clearly inviable.

To measure the impact of temperature on larval life, we maintained the tadpoles in the same thermal conditions under which they had been kept as eggs. To do so, we combined all the newly hatched tadpoles from the same temperature treatments, and then randomly selected 30 tadpoles from each of these groups to allocate to each replicate container (i.e., all tadpoles in a container were from the same egg‐temperature treatment, which in turn was the same as the larval temperature treatment experienced by those animals). As before, we used six replicate containers per temperature treatment. We maintained constant densities of tadpoles in each container (30 tadpoles per tub) to avoid variable‐density effects on rates of survival and growth of tadpoles (Browne, Pomering, & Hamer, 2003; Cameron, 1994). The “reserve” tadpoles were maintained in the laboratory under identical temperatures and container sizes as the treatment animals, and added only if needed to maintain densities in the experimental containers. The tadpoles were fed with frozen lettuce daily (ad libitum), and the water was changed once a week. Ten randomly selected tadpoles per container were photographed (Canon, IXUS 115 HS, Canon Australia) weekly, so that we could measure total lengths accurately using ImageJ software (Rasband, 2012). Weekly counts of tadpole numbers per tub gave direct measures of the rate of survival.

After tadpoles reached stage 40, they were weighed daily (Tanita, Model 1478, Arlington Heights, Illinois; ±0.01 g) to quantify maximum body mass at the onset of metamorphosis. Mass and snout–urostyle length (SUL) of each metamorph were recorded also (at stage 42, when the forelimbs appeared), and metamorphs were photographed for later measurement and analysis of morphometrics. The number of days elapsed between Gosner stages 25 and 42 was used as our measure of age at metamorphosis. After they reached Gosner stage 42 (forelimbs emerged), the metamorphs were then raised in tubs (39 × 28 × 14 cm; half water, half sand; each containing 20 metamorphs from the same thermal treatment) and fed with one‐day‐old crickets thrice weekly.

One month postmetamorphosis, toads were tested for their absolute speed (cm/s), relative speed (body lengths/s), and distance per hop. Metamorphs were again photographed for additional morphometric traits (intraorbital distance, SUL, tibia length). The locomotor performance of metamorphs was measured using a 1‐m racetrack, with toadlets encouraged to move by gently tapping the urostyle with a soft brush. When the toad reached the end of the raceway, it was turned around and encouraged to hop back to the opposite end. This process was continued until the toadlet refused to respond to being touched with the brush for 1 min (our criterion for exhaustion). Endurance was calculated by dividing the total distance traveled by each toadlet by its SUL. We calculated speeds over two distances: the total distance traveled by each toadlet, and over the first 1 m. Our data on body lengths per hop were obtained by dividing the total distance traveled by the number of hops used. Each toad metamorph was tested only once.

### 2.4 Statistical analysis

We used SPSS version 20 (IBM, Armonk, New York) for statistical analysis. All tests were performed with *α* < .05. Analysis of variance (ANOVA) was performed to compare mean pond temperatures between seasons (spring vs. autumn). We used a general linear model with log link function to assess the effect of water temperature on hatching success of the eggs. Where thermal effects were significant, we performed multiple pairwise comparisons (Tukey's pairwise tests) to locate the significance more precisely. Clutch identity was included as a random factor in all analyses of egg and larval variables. To quantify survival rates of tadpoles at each test temperature, we combined successive weekly estimates of survival. That is, we estimated survival in Week 1, Week 2, etc., independently, and then combined these estimates (i.e., successively multiplied the % survival at the beginning of that week [from the time of hatching] by the % survival recorded during that week) to quantify overall rates of survival from hatching to metamorphosis. The reason for this approach was that we replaced any tadpoles that died during the experiment; not to do so would have confounded temperature effects with density effects (e.g., a specific temperature might cause high initial mortality, meaning that the survivors thereafter experienced low densities and as a result of lower conspecific competition, grew faster and survived better). By replacing dead tadpoles, we maintained constant densities, and thus, any difference between treatments is due to water temperature not to variable densities. The focus of our study is overall viability, so we simply compared the proportion of tadpoles surviving to metamorphosis among treatments using ANOVA with treatment as the factor (rather than examining the time course of mortality in different treatments using survival analyses). When results were significant, post hoc Tukey's HSD tests were used to identify the location of significant differences among treatments. Rates of tadpole growth as well as survival were analyzed using mean values per container (because we were unable to recognize individual tadpoles).

Analysis of variance was performed to examine the effects of temperature on maximum tadpole body mass reached before metamorphosis, age at metamorphosis, and metamorphic mass. Analysis of covariance (ANCOVA), with SUL as the covariate, was used to assess treatment effects on metamorph body size, morphology (body condition, relative dimensions of the head and limbs), and toadlet jumping ability (absolute speed, endurance, hopping speed, and length per hop). When the results were significant, we performed pairwise comparisons among treatments. Analyses of maximum body mass of tadpoles, metamorphic mass of tadpoles, toadlet morphology, and jumping performance were based on data from individual animals. To avoid pseudoreplication, we included container number and clutch identity as random factors in the analyses of tadpole data. High mortality at metamorphosis resulted in only one or two containers of surviving metamorphs from each treatment (pooled from previous larval containers; one clutch per container), so we only included clutch identity as a random factor in analyses of metamorph morphology and performance.

## 3 Results

### 3.1 Water temperatures in spawning ponds

**Figure 2:**
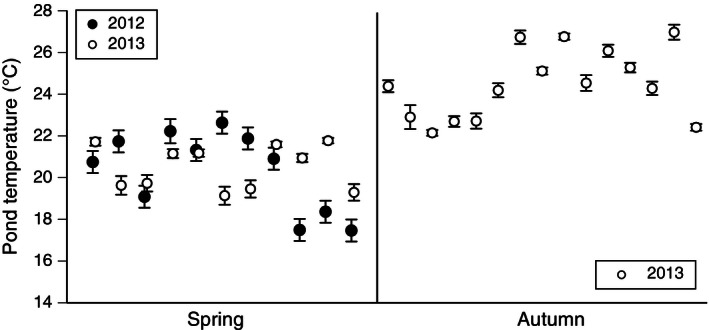
Variation of pond temperature among seasons. Each data point shows the mean and standard error of water temperature as measured over a five‐day period in a single pond.

Water temperatures in spring averaged 20.19–20.58°C, whereas those in autumn averaged 24.25–24.88°C (Fig. 2). Broadly, mean pond temperatures varied among seasons, differed between successive years (even in the same pond at the same time of year), and varied among adjacent ponds tested at the same time (Fig. 2). To establish biologically realistic temperatures at which to test toad eggs and larvae in the laboratory, we selected the range from 16°C (slightly below the coolest pond measured in spring; Fig. 2) to 32°C (the mean recorded in tropical spawning ponds used by this species; Hagman & Shine, 2006).

### 3.2 Effects of temperature on hatching success of eggs

**Figure 3:**
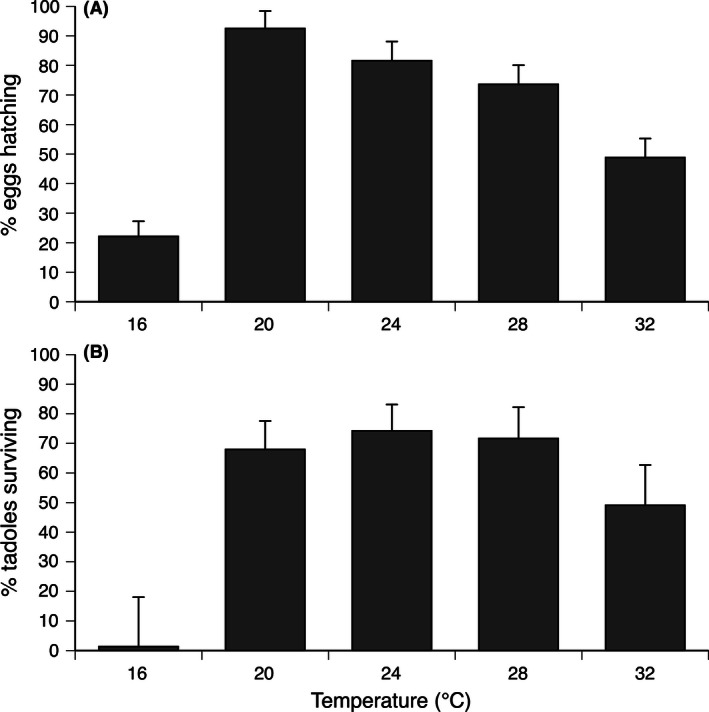
The effects of temperature treatment on (A) hatching success of eggs and (B) the overall survival of tadpoles between hatching and metamorphosis. The figure shows mean values and associated standard errors

Hatching success of eggs was lowest at 16°C, highest at 20°C, and then declined as temperature increased to the highest temperature we used (32°C). Overall, water temperature significantly affected the percentage of eggs that hatched (*F*_4,24_ = 25.45, *p *<* *.0001; Fig. 3A).

### 3.3 Effects of temperature on the survival of tadpoles

The rate of tadpole survival through to metamorphosis was highest at intermediate water temperatures (*F*_4,22_ = 4.54, *p *<* *.011; Fig. 3B). Post hoc Tukey's HSD tests show that survival to metamorphosis was significantly lower at 16°C than at any other temperature (except 32°C).

### 3.4 Effects of temperature on the growth rate of tadpoles

**Figure 4:**
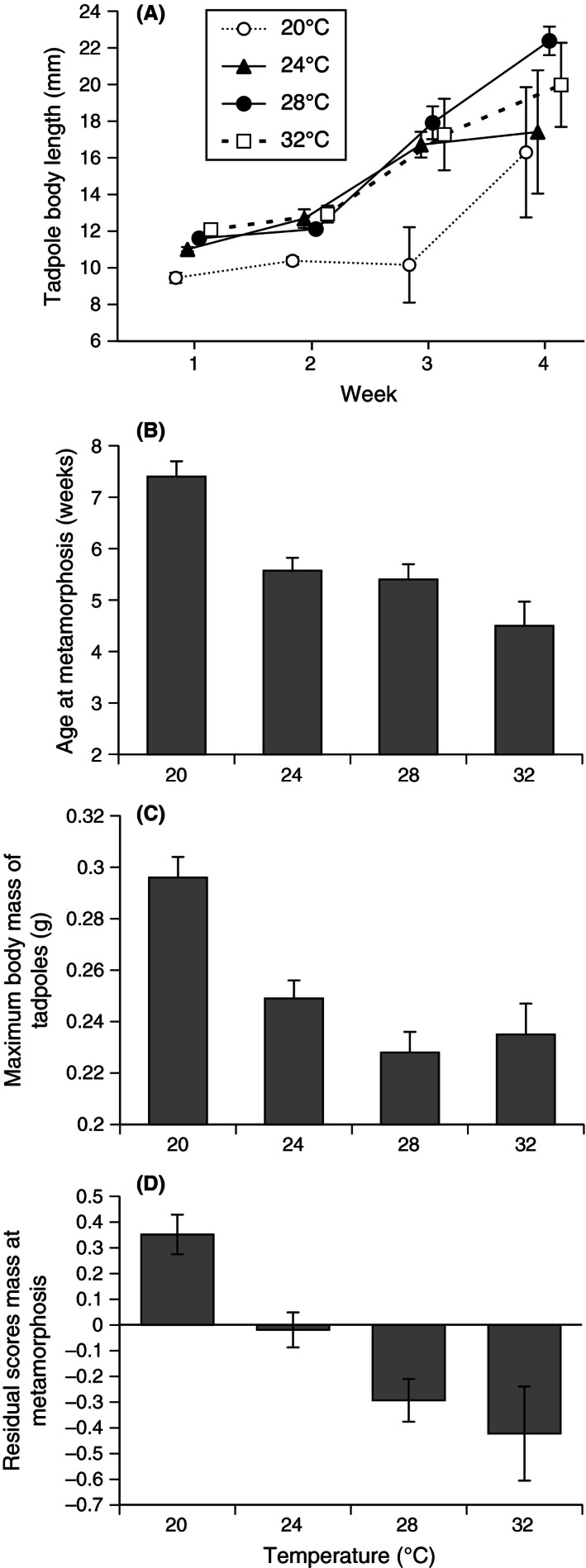
Effect of temperature treatment on (A) body lengths of tadpoles through time, (B) age at metamorphosis, (C) maximum body mass of tadpoles, and (D) mass at metamorphosis relative to snout–urostyle length (SUL; residual scores from linear regression of ln‐transformed values of mass vs. SUL). The figure shows mean values and associated standard errors

Water temperature affected the rate of growth of tadpoles (repeated‐measures ANOVA, *F*_3,16_ = 6.53, *p *=* *.004), with faster growth at higher temperatures. However, the slower growth of cool‐water tadpoles (20°C) was evident only for the first 3 weeks; by the fourth week, these tadpoles were almost as large as their siblings raised at higher temperatures (Fig. 4A).

### 3.5 Effects of temperature on age and size at metamorphosis

Tadpoles from low temperature (20°C) metamorphosed later than did their siblings from high temperature (ANOVA, *F*_3,15_ = 12.98, *p *<* *.0001; Fig. 4B). Water temperature also affected the maximum body mass reached by tadpoles prior to metamorphosis (ANOVA, *F*_3,15_ = 14.47, *p *<* *.0001; Fig. 4C), as well as mass at metamorphosis (ANOVA, *F*_3,15_ = 3.53, *p *=* *.04). Metamorphs from a low‐temperature (20°C) treatment tended to have higher body condition than those from other treatments (metamorphic mass relative to body length: ANCOVA, *F*_3,516_ = 16.59, *p *<* *.0001; Fig. 4D). Heavier tadpoles became heavier metamorphs (ANOVA, *F*_1,14_ = 5.15, *p *=* *.04), and there was a significant interaction between temperature, maximum body mass of tadpoles, and mass at metamorphosis (ANOVA, *F*_3,11_ = 5.26, *p *=* *.001).

### 3.6 Effects of temperature on the morphology and locomotor ability of metamorphs

**Figure 5:**
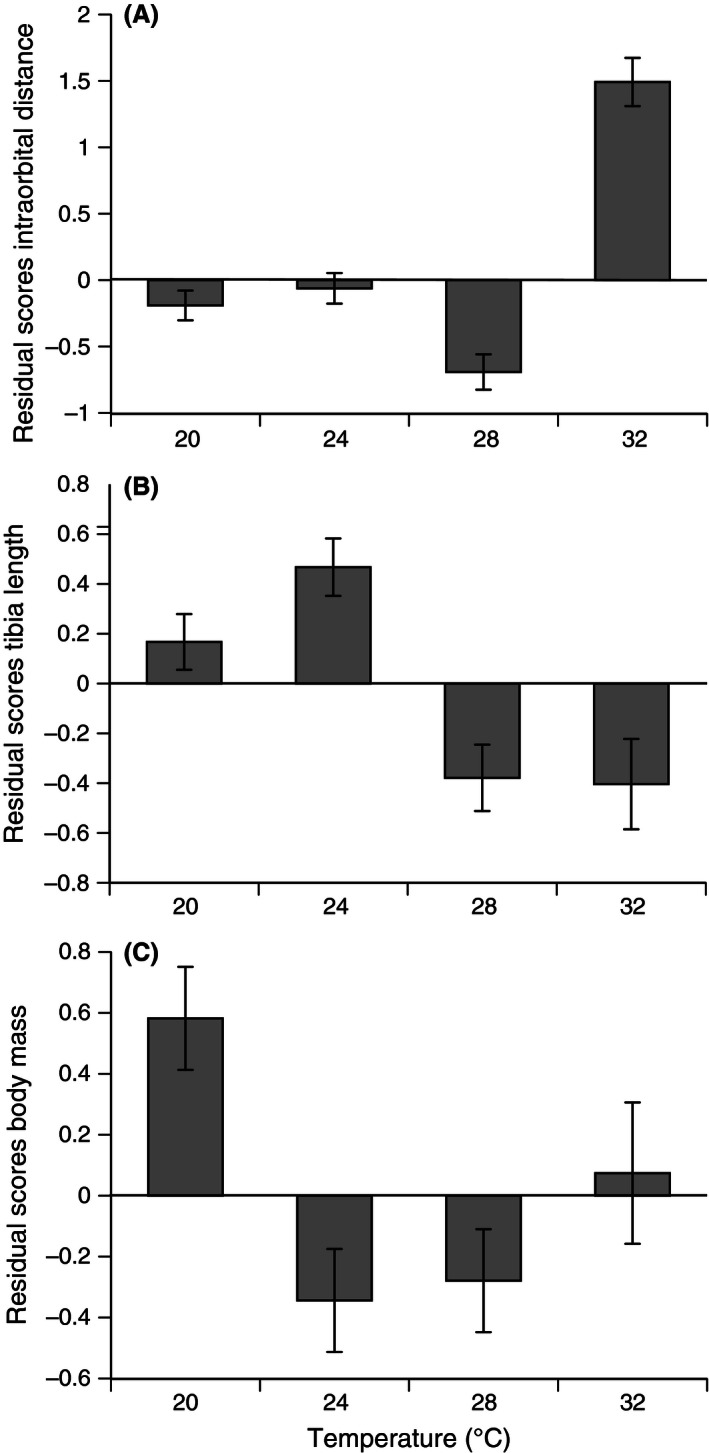
Effect of temperature treatment on body size and shape of postmetamorphic cane toads. The panels show residual scores from linear regressions of ln‐transformed values of the morphological trait vs. snout–urostyle length (SUL). (A) Intraorbital distance (IOD) = IOD relative to SUL; (B) tibia length = tibia length relative to SUL; and (C) body mass = body mass of toadlets relative to SUL. The figure shows mean values and associated standard errors

Toadlets from all treatment temperatures were similar in body length (SUL: ANOVA, *F*_3,105_ = 2.09, *p *=* *.11). Water temperature affected toadlet body shape, with warmer water resulting in metamorphs with broader heads relative to SUL (ANCOVA, *F*_3,104_ = 41.18, *p *<* *.0001; post hoc Tukey's HSD shows 32°C > 24°C = 20°C > 28°C; see Fig. 5A) and shorter tibias relative to SUL (ANCOVA, *F*_3,104_ = 5.57, *p *<* *.001; post hoc Tukey's HSD shows 20°C > 28°C; Fig. 5B). Cooler water resulted in toadlets with higher body condition (ANCOVA, *F*_3,101_ = 6.54, *p *<* *.0001; post hoc Tukey's HSD shows 20°C = 32°C > 24°C = 28°C; see Fig. 5C).

**Figure 6:**
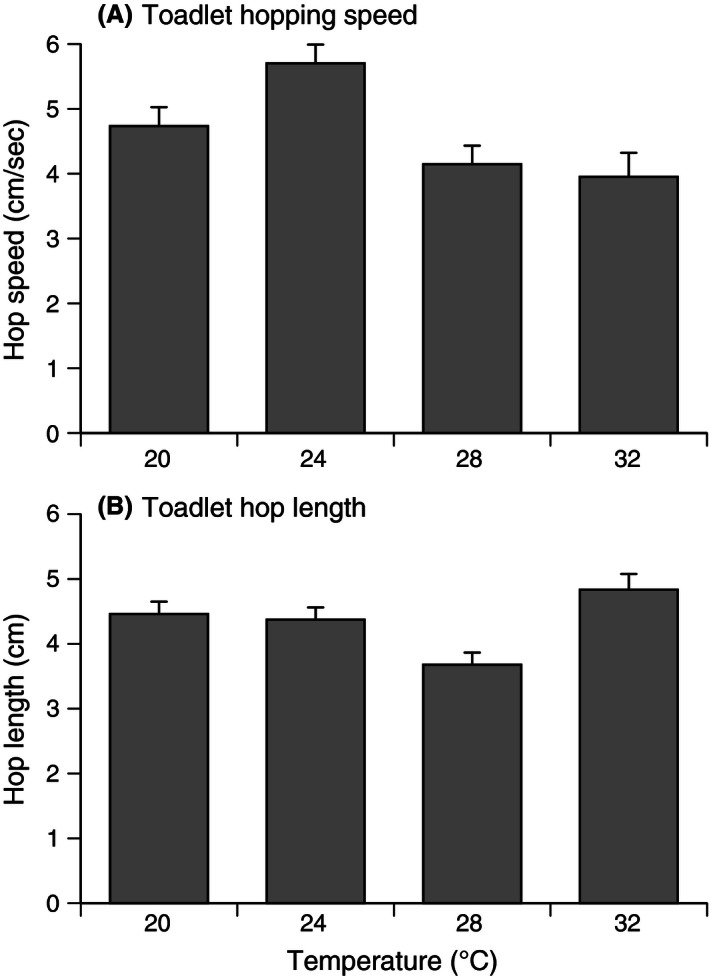
Effect of temperature treatment on jumping ability of metamorphic cane toads. (A) Hopping speed = distance traveled (per second); and (B) hop length = length per jump. The figure shows mean values and associated standard errors

Despite these morphological effects, rearing temperature did not modify the toadlets’ absolute speed relative to body size over the total distance they traveled during a trial (ANCOVA, *F*_3,103_ = 2.25, *p *=* *.08). However, rearing temperature affected speed relative to body size over the first 1 m traveled (ANCOVA, *F*_3,103_ = 6.69, *p *<* *.0001; post hoc Tukey's HSD shows 24°C > all other treatments; see Fig. 6A) and body lengths per hop (ANCOVA, *F*_3,103_ = 5.61, *p *=* *.001; post hoc Tukey's HSD shows 32°C = 20°C > 28°C; see Fig. 6B); toadlets from 24°C were faster, and toadlets from 32°C took longer hops.

## 4 Discussion

The cane toad invasion front is gradually moving southward in NSW, primarily through a narrow coastal corridor between the Pacific Ocean and the Great Dividing Range. In this region, abiotic conditions in spawning ponds differ from those available within the species’ native range in terms of pH, salinity, and temperature (Evans, Yáber, & Hero, 1996; Lever, 2001). Therefore, we might expect these extreme abiotic conditions to prevent toads from extending their range further south. However, our studies falsify this prediction. In other work, we have shown that neither pH nor salinity levels in this area are likely to prevent successful reproduction by cane toads (Wijethunga, Greenlees, & Shine, 2015, 2016). A third possibility, addressed in the current study, is that lower temperatures will constrain toad reproduction in more southerly regions. In keeping with that idea, pond temperatures at our study sites in spring and autumn were lower (20–25°C) than have been reported in water bodies used for breeding within the species’ native range in Venezuela (29.2–29.9°C; Evans et al., 1996) and tropical Australia (31–32°C; Hagman & Shine, 2006). In a study conducted closer to the current work, Semeniuk et al. (2007) reported midsummer water temperatures of 26.8–28.4°C. Our laboratory experiments, based on toads collected close to the current southern edge of the species’ range, confirm that several aspects of the early life history are highly sensitive to ambient water temperatures. However, the nature of those effects suggests that cane toads are capable of reproducing successfully in conditions cooler than exist at the species’ current range limit (even without considering the likely increase in water temperatures due to global climate change; Ficke, Myrick, & Hansen, 2007).

Given the tropical origin of *Rhinella marina*, and records of high water temperatures in spawning ponds within its native range (Evans et al., 1996), we expected that eggs and larvae would exhibit higher viability under warmer conditions. Our data do not support that prediction. Higher temperatures accelerated larval development, which might enhance fitness if earlier metamorphosis increases survival (as would occur, e.g., if water bodies often dried out before toads could metamorphose). In most other respects though, eggs and larvae thrived at least as well in cool water (down to 20°C) as in warmer conditions (up to 32°C). Water temperature significantly affected hatching success, tadpole survival, tadpole length, and metamorphic mass and locomotor ability. Adult toads from NSW populations similarly are able to function effectively at lower temperatures than they experience over most of their native range (McCann et al., 2014). The ability of these ancestrally tropical anurans to thrive at temperatures lower than usually experienced in their native range (e.g., Fig. 3) is surprising, and may reflect rapid adaptation to the cooler conditions prevailing in southern Australia. Comparable studies on thermal tolerances of toads in tropical Australia would be of great interest.

The thermal sensitivity exhibited by cane toad larvae is widespread in anurans. In many species, water temperature determines the rate of development, as well as affecting larval behavior (Moore & Townsend, 1998), developmental and growth rate (Berven & Gill, 1983; Berven, Gill, & Smith‐Gill, 1979; Gomez‐Mestre & Buchholz, 2006; Moore, 1939), and survival and reproductive success (Berven, 1982; Harkey & Semlitsch, 1988; Smith, 1987). A previous study reported that cane toad embryos can tolerate temperatures from 17 to 34°C and larvae can develop between 20 and 34°C, with little impact of temperature on larval survival over that range (Floyd, 1983). However, Floyd's (1983) studies were based on toads from tropical Queensland. Given the strong geographic divergence in morphology, physiology, and behavior among Australian populations of cane toads (Alford, Brown, Schwarzkopf, Phillips, & Shine, 2009; Brown et al., 2015; Llewelyn et al., 2010; Phillips et al., 2006), studies on cold tolerance need to be based on specimens from local populations at the southern (cool‐climate) limits of the species’ current Australian range.

The mechanistic basis and selective consequences for the kinds of effects we documented have been discussed by previous studies. Lower survival of tadpoles at high temperature may be due to a higher metabolic rate and reduced metabolic efficiency under these conditions (Packard & Packard, 1988). The trend for cooler temperatures to increase body size at metamorphosis is widespread in ectotherms (Atkinson, 1996; Sibly & Atkinson, 1994). Low temperature appears to retard differentiation more than growth rate, thereby increasing stage‐specific size (Smith‐Gill & Berven, 1979). In terms of adaptive significance, low temperatures may incur both costs and benefits. Costs include compromised physiological functions such as feeding rate and predator avoidance, due to reduced metabolic rates (Broomhall, 2004). Slower larval development increases exposure to predators and the risk of pond desiccation (Newman, 1988; Relyea, 2007), both of which are major causes of larval mortality in amphibians (Newman, 1987; Smith, 1983). Benefits revolve mostly around larger size at metamorphosis, which can enhance fitness via increased terrestrial performance (Pough & Kamel, 1984), higher juvenile survivorship (Martof, 1956), larger size at maturity (Berven, 1982; Smith, 1987), and better reproductive success (Hetherington, 1988; Newman & Dunham, 1994; Pough & Kamel, 1984). In cane toads, studies in outdoor enclosures confirm that large body size at metamorphosis is likely to enhance an individual's long‐term viability (Cabrera‐Guzmán, Crossland, Brown, & Shine, 2013). Laboratory studies show that larger body size in metamorphic toads reduces mortality due to parasitic lungworms (Kelehear et al., 2009), predation by cannibalistic conspecifics (Pizzatto & Shine, 2008) and other predator species (Greenlees, Phillips, & Shine, 2010; Ward‐Fear, Brown, & Shine, 2010), and desiccation (Child, Phillips, Brown, & Shine, 2008; Child, Phillips, & Shine, 2008). Mathematical models of toad recruitment suggest that size at metamorphosis may be a key contributor to rates of population growth (Beaty & Salice, 2013).

The impacts of water temperature during larval life can extend to postmetamorphic morphology and performance. In our study, warm water (32°C) resulted in toadlets with relatively short legs and broad heads, and with lower body mass relative to SUL. Those morphological effects were accompanied by an increased locomotor speed of cool‐developing toadlets, at least over the first meter of the racetrack. Size at metamorphosis correlates positively with sprint speed, stamina, jumping ability, and endurance in other *Bufo* species (Beck & Congdon, 2000; Goater, Semlitsch, & Bernasconi, 1993; John‐Alder & Morin, 1990). The toadlets from warm water achieved greater body lengths per hop, despite the fact that work on other anurans has reported a locomotor advantage to longer limbs (Emerson, 1978).

Clearly, there are limits to thermal tolerance. In our experiments, toad eggs hatched successfully over the range from 20 to 32°C, but success was lower at 16°C (our lowest temperature). Similarly, Floyd (1983) reported cane toads spawning at water temperatures from 17 to 28°C, but spawn at 17–18°C failed to develop. In combination, the available information thus suggests a thermal threshold of around 19°C for successful breeding of cane toads. Field studies are needed to identify how that threshold will translate into areas suitable for cane toads in southern Australia. We predict that the toads can breed successfully in any area where pond temperatures exceed 18°C for at least 2 months per year. That condition is likely to be fulfilled well south of the current invasion front. In keeping with that prediction, a stowaway‐founded population of toads bred successfully in Sydney (around 600 km south of the main range edge) in at least five successive years (M. Greenlees, unpubl. data). The ability to rapidly adapt to low temperatures might contribute to the higher adult density of cane toads in Australia than in their native range (Lampo & De Leo, 1998).

What do our results suggest about the ultimate limits to cane toad invasion in temperate Australia? It is difficult to predict population‐level responses to changes in traits such as hatching success, larval duration, and size at metamorphosis, because of complex feedback loops and nonlinearities in the links between life‐stage parameters and subsequent population size (Beaty & Salice, 2013; Lampo & De Leo, 1998). Nonetheless, our data can provide a basis for more robust estimates than has heretofore been possible. Previous models used to predict the lower thermal limits for toad distribution typically have relied upon conditions within the native range (or already‐invaded range as well) or have focused on biology of postmetamorphic toads (e.g., Kearney et al., 2008). Our experimental results suggest that the current southern limit of cane toad distribution in Australia is still well above their critical thermal limits for successful reproduction. Ideally, future modeling efforts should consider both larval and adult tolerance limits to environmental conditions (and plasticity in those tolerances; Kolbe, Kearney, & Shine, 2010; McCann et al., 2014) to more accurately forecast range limits. Importantly, we need information on toads from a range of localities within Australia, because the thermal tolerances of local populations may well differ (as suggested by the contrast between our results and those based on tropical conspecifics; Floyd, 1983). The dynamic and rapidly evolving traits of invasive species (Carroll, Klassen, & Dingle, 1998; Whitney & Gabler, 2008) add substantial complexity to any attempt to predict invader distributions.

## Conflict of Interest

None declared.

## Funding Information

Australian Research Council (Grant/Award Number: FL120100074)
